# User-Centered Development of STOP (Successful Treatment for Paranoia): Material Development and Usability Testing for a Digital Therapeutic for Paranoia

**DOI:** 10.2196/45453

**Published:** 2023-12-08

**Authors:** Che-Wei Hsu, Daniel Stahl, Elias Mouchlianitis, Emmanuelle Peters, George Vamvakas, Jeroen Keppens, Miles Watson, Nora Schmidt, Pamela Jacobsen, Philip McGuire, Sukhi Shergill, Thomas Kabir, Tia Hirani, Ziyang Yang, Jenny Yiend

**Affiliations:** 1 Department of Psychological Medicine Dunedin School of Medicine University of Otago Dunedin New Zealand; 2 Department of Psychosis Studies King's College London Institute of Psychiatry, Psychology & Neuroscience London United Kingdom; 3 Department of Biostatistics and Health Informatics King's College London, Institute of Psychiatry, Psychology & Neuroscience London United Kingdom; 4 University of East London London United Kingdom; 5 Department of Psychology King's College London Institute of Psychiatry, Psychology & Neuroscience London United Kingdom; 6 South London and Maudsley National Health Service Foundation Trust London United Kingdom; 7 Department of Informatics King's College London London United Kingdom; 8 University of Bath Bath United Kingdom; 9 Department of Psychiatry University of Oxford Oxford United Kingdom

**Keywords:** cognitive bias modification, paranoia, content specificity, mental health, mobile app, mhealth, digital therapeutic, user-centered development, user, user-friendly app, paranoid, persecution, persecution complex, delusions, obsession, megalomania, monomania, psychosis, psychotic

## Abstract

**Background:**

Paranoia is a highly debilitating mental health condition. One novel intervention for paranoia is cognitive bias modification for paranoia (CBM-pa). CBM-pa comes from a class of interventions that focus on manipulating interpretation bias. Here, we aimed to develop and evaluate new therapy content for CBM-pa for later use in a self-administered digital therapeutic for paranoia called STOP (“Successful Treatment of Paranoia”).

**Objective:**

This study aimed to (1) take a user-centered approach with input from living experts, clinicians, and academics to create and evaluate paranoia-relevant item content to be used in STOP and (2) engage with living experts and the design team from a digital health care solutions company to cocreate and pilot-test the STOP mobile app prototype.

**Methods:**

We invited 18 people with living or lived experiences of paranoia to create text exemplars of personal, everyday emotionally ambiguous scenarios that could provoke paranoid thoughts. Researchers then adapted 240 suitable exemplars into corresponding intervention items in the format commonly used for CBM training and created 240 control items for the purpose of testing STOP. Each item included newly developed, visually enriching graphics content to increase the engagement and realism of the basic text scenarios. All items were then evaluated for their paranoia severity and readability by living experts (n=8) and clinicians (n=7) and for their item length by the research team. Items were evenly distributed into six 40-item sessions based on these evaluations. Finalized items were presented in the STOP mobile app, which was co-designed with a digital health care solutions company, living or lived experts, and the academic team; user acceptance was evaluated across 2 pilot tests involving living or lived experts.

**Results:**

All materials reached predefined acceptable thresholds on all rating criteria: paranoia severity (intervention items: ≥1; control items: ≤1, readability: ≥3, and length of the scenarios), and there was no systematic difference between the intervention and control group materials overall or between individual sessions within each group. For item graphics, we also found no systematic differences in users’ ratings of complexity (*P*=.68), attractiveness (*P*=.15), and interest (*P*=.14) between intervention and control group materials. User acceptance testing of the mobile app found that it is easy to use and navigate, interactive, and helpful.

**Conclusions:**

Material development for any new digital therapeutic requires an iterative and rigorous process of testing involving multiple contributing groups. Appropriate user-centered development can create user-friendly mobile health apps, which may improve face validity and have a greater chance of being engaging and acceptable to the target end users.

## Introduction

### Background

Psychosis is one of the most disabling mental health conditions presenting with significant distress, suicidal ideation, impaired social and occupational functioning, and physical ill-health [1,2]. Paranoia and associated delusions are common symptoms of psychosis, are associated with more distress than other types of delusion [3], are most likely to be acted upon [4], and represent a strong predictor of hospitalization [5]. In the United Kingdom, over one-third of patients with psychiatric conditions experience paranoia, which also presents in a range of other psychopathologies such as depression [6], bipolar disorder [7], posttraumatic stress disorder [8], anxiety [9], as well as schizophrenia [10].

The National Institute for Health and Care Excellence recommended cognitive behavioral therapy (CBT) for treating psychosis. CBT, however, is received by only 1 in 10 of those who could benefit and has shown only moderate effect sizes for the treatment of delusions [11,12], although effect sizes are higher for those studies targeting delusions specifically, as opposed to generic CBT [13,14]. Unfortunately, a significant proportion of patients having paranoia continue to experience distressing symptoms following psychological treatment [15,16]. Consequently, there is a need for novel, highly accessible, and low-cost interventions for paranoia, either as standalone treatments or as adjuncts to boost existing therapies. Cognitive bias modification (CBM) is a class of intervention that may address these needs.

### Cognitive Bias Modification

The class of CBM interventions works on the premise that cognitive bias is a putative causal factor of various mental health concerns [17-21]. One form of cognitive bias is interpretation bias, which is the tendency for individuals to think about a situation in a negatively skewed direction. However, the same situation could also be interpreted in a benign or positive direction. Repeated negatively biased interpretations are thought to contribute to the formation and maintenance of psychological symptoms and increase distress [3]. Across many studies, researchers have found evidence of interpretation bias among anxiety [19], depression [20], and social phobia [21], with some work on interpretation bias in paranoia [3,22-25].

CBM is a class of targeted treatment that focuses on manipulating naturally occurring interpretation bias in a more helpful direction, with findings from many studies demonstrating the positive efficacy of CBM with various psychiatric disorders, including anxiety, affective disorders, and substance addictions [26-28]. There are several benefits to CBM. First, CBM can be self-administered and disseminated over numerous settings [29], thereby reducing the need for mental health professionals. Next, CBM has the potential to benefit patients whose symptoms may influence their trust in a therapist [30]. Third, CBM can be delivered on a digital platform, which means that it is highly accessible at a low cost [31,32].

**Figure 1:**
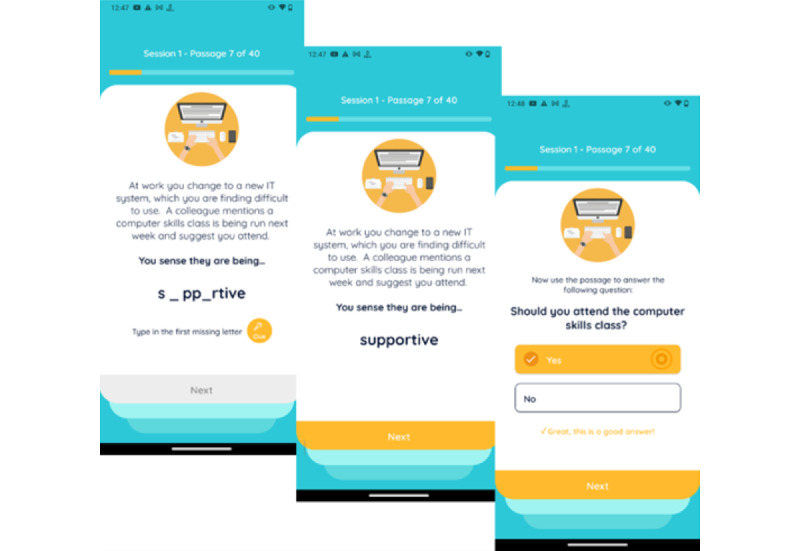
Example of a STOP intervention item. Copyright © 2021. Jenny Yiend, King's College London. All rights reserved.

Despite these benefits and the positive efficacy of CBM with various mental health concerns, there is a dearth of studies on CBM that address psychosis, with only some preliminary evidence of the feasibility and implications of this approach. For example, Steel et al [33] demonstrated the effects of CBM on anxiety in individuals diagnosed with schizophrenia. The results from that study showed that a subgroup of participants exhibited positive changes in interpretation bias. Turner et al’s [34] case study on patients who experienced social anxiety following a psychotic episode demonstrated similar positive changes in interpretation bias. In a feasibility study, Yiend et al [35] directly examined the effects of CBM in patients with paranoia, using an intervention called CBM for paranoia (CBM-pa). In that study, 63 participants with clinically significant persecutory or paranoid symptoms were randomly assigned to either the CBM-pa group (n=32) or the control group (n=31). Participants in the CBM-pa group were presented with 40 short passages over 6 weekly sessions using a software called E-prime (Psychology Software Tools, Inc). Users were invited to complete the final word of each passage, which contained missing letters. Once completed, the word resolved the ambiguity of the passage in a benign nonparanoid manner. A follow-up yes or no question reinforced the benign interpretation of the passage (see Figure 1). The sessions were self-directed as users completed each word task independently on the computer. The control group received the same number of sessions over 6 weeks that included items of general knowledge and facts and everyday activities. Results showed that relative to the control group, participants in the CBM-pa group showed larger reductions in negative interpretation bias and paranoid symptoms.

Each passage of CBM-pa depicted an emotionally ambiguous scenario, all of which were developed with a user-centered approach, by inviting living experts and experienced clinicians to review all training materials to ensure the clinical relevance of the items to paranoia.

### User-Centered Development

Researchers have shown that people experiencing psychosis can benefit from digital therapeutics, but despite the wide availability of digital therapeutics on the app market, many have insufficient evidence-based data to support their efficacy, design, and development [36]. It is important to take a user-centered development approach to design user-friendly, engaging, and self-managing digital therapeutics for psychosis [37,38] by involving multiple collaborators, including service users, researchers, and the design team. This approach is known to increase the adoption of the app by end users [38] and improve app design and content [39,40]. Self-administered mobile health apps without quality evidence-based data to support their use may decrease the usability and effectiveness of the treatment [41]. This is important for both app design as well as the intervention content. Researchers have demonstrated that biases are stronger when the encountered situation aligns with the individual's common everyday experiences [42,43]. Yiend et al [35] used content-specific training materials for paranoia to capture and modify paranoia interpretation bias commonly experienced by patients with paranoid symptoms. Content materials were co-designed with relevant contributors, and sessions were presented in rank order of increasing severity of items using Freeman et al’s [44] hierarchy of paranoia as a guide. The training items covered 6 categories relevant to paranoia: social or interpersonal threat, delusions of reference or magical thinking, the threat of persecution or spying, general suspiciousness or distrust, medical or paramedical or health care threat, and physical harm.

### This Study

Building on from Yiend et al [35] and following a user-centered development approach, we aimed to develop CBM-pa into a 12-session mobile app therapeutic called STOP (Successful Treatment of Paranoia). As a part of an ongoing clinical trial, we are testing STOP’s efficacy, we tested STOP’s efficacy against the control group. STOP included the original item content from the CBM-pa feasibility study and newly developed items for 6 additional training sessions (details of content development for the 6 training sessions from the CBM-pa feasibility study will be reported separately). In this paper, we reported the detailed development process of STOP, which had the following objectives: (1) take a user-centered approach with input from living or lived experts, clinicians, and academics to create and evaluate paranoia-relevant item content to be used in STOP and (2) engage with living or lived experts and the design team from Avegen to cocreate and pilot-test the STOP mobile app prototype. Avegen is a digital health care company specializing in developing innovative health care technologies [45].

The methodology of the STOP development process involved (1) 4 stages for objective 1: text creation, text evaluation, graphics development, and graphics evaluation and (2) 1 stage for objective 2—STOP mobile phone app usability testing.

Objective 1 was intended to ensure clinical relevance, content specificity to paranoia, face validity of the training materials, and user acceptability for STOP. Objective 2 provided data on living or lived experts’ perspectives on the functionality, interface, and acceptability of the prototype STOP app to reveal areas of strength and those that needed improvement.

### STOP Content Design (Test and Images)

**Figure 2:**
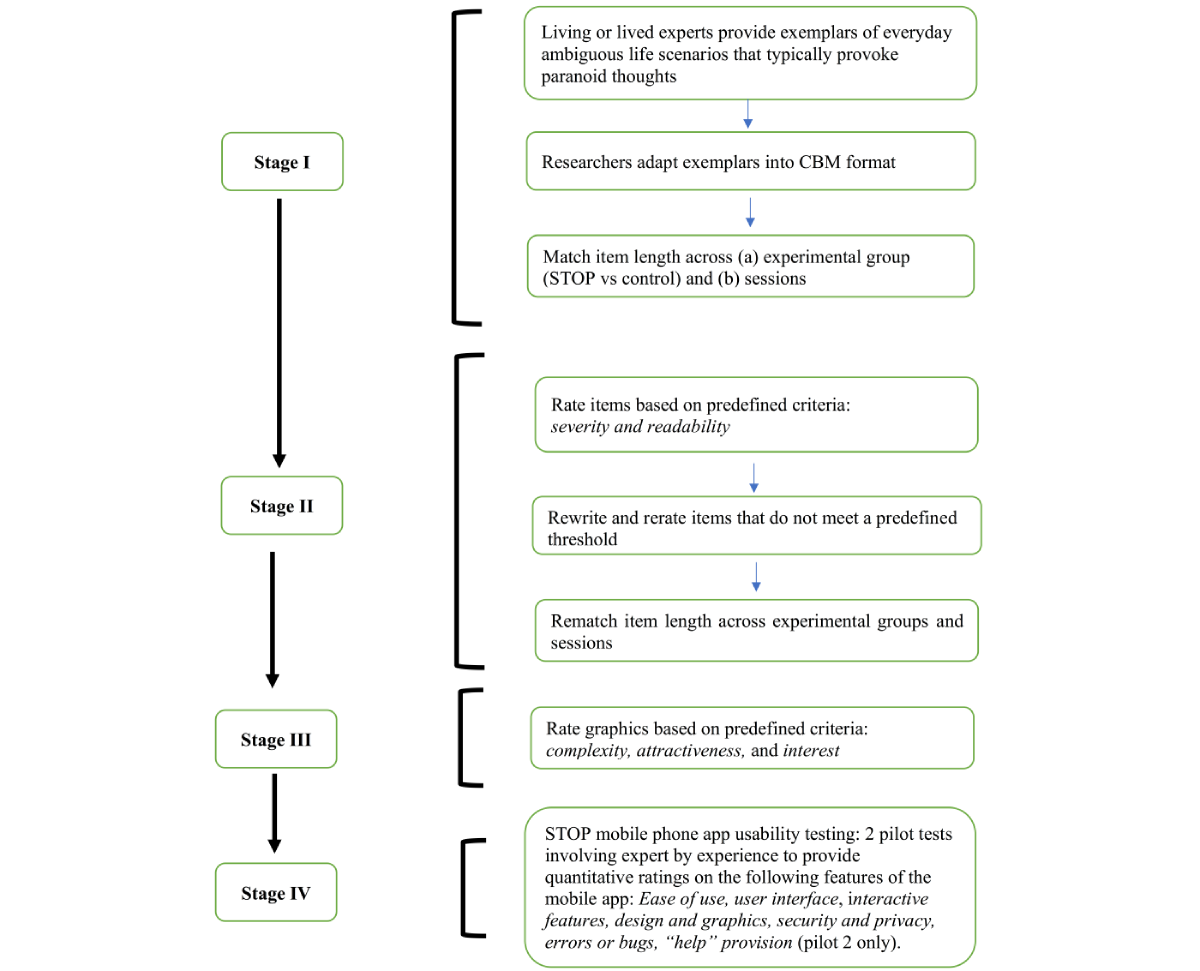
Schematic representation of the development process of STOP materials.

The process of material development and testing spanned 12 months and involved extensive iterative input from (1) living or lived experts, (2) clinical psychologists, and (3) the STOP academic research team (see Multimedia Appendix 1 [46,47] for inclusion criteria of each contributor). Input from each contributing group and the numbers varied according to the task required. Figure 2 shows a schematic of the development process.

### Stage I: Scenario Creation

#### Introduction

To improve the content specificity of training materials, which has been shown to better capture disorder-specific biases [42,43], living or lived experts were invited to generate CBM materials for paranoia based on their common everyday experiences. We aimed to adapt user-generated scenarios into CBM intervention items.

#### Methods

##### Participants

Living or lived experts (n=18) were recruited from the Lived Experience Advisory Panel (LEAP) and wider networks with the help of a coauthor (TK) from the McPin Charity Foundation—an organization based in the United Kingdom that focuses on championing lived experience expertise in mental health research [48]. McPin collaborates with living or lived experts to invite their feedback in research. Experts were reimbursed for their contribution to this study at £30 (US $36.67) per hour.

##### Scenario Creation Outline

We provided our living or lived experts with written information on CBM and guidelines in addition to examples for creating exemplars of personal everyday life scenarios that could provoke paranoid thinking (see Multimedia Appendix 1 for a full description).

#### Results

##### Intervention Items

The STOP research team adapted suitable scenarios (excluding items that were too bizarre, triggering, or did not capture ambiguity) into 240 intervention items in the format commonly used for CBM training items (see Figure 1). Each item consists of 3 lines of text depicting an emotionally ambiguous scenario that could be either interpreted as paranoid or nonparanoid. The item remains ambiguous until the final word. The final word contains missing letters and is used to resolve the scenario in a nonparanoid manner. One or more letters (depending on the length of the final word) are removed from the final word (in some items this encompasses the last 2-3 words).

##### Text-Reading Control

**Figure 3:**
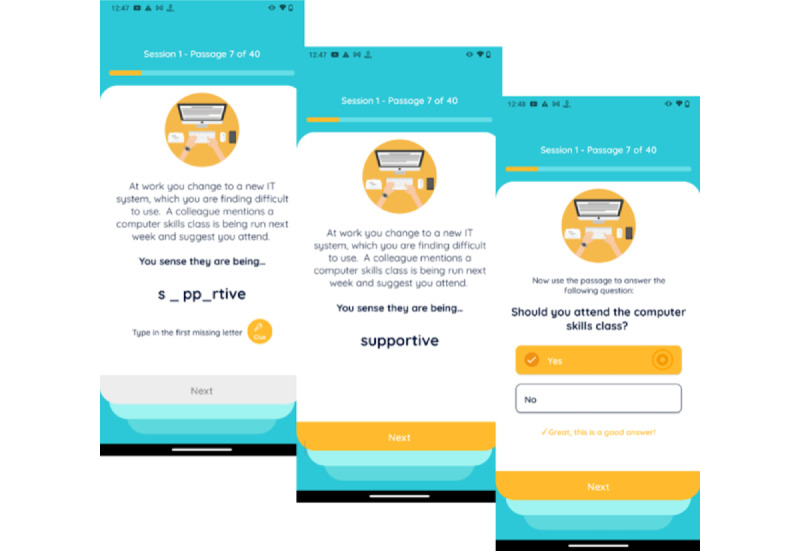
Example of a STOP control item. Copyright © 2021. Jenny Yiend, King's College London. All rights reserved.

In total, 240 control items were created based on nonemotional factual information or mundane activities or sequences of actions (eg, making a cup of tea). The control items excluded depictions of social situations, emotional words, and feelings. Items were arranged into 2 topic areas or categories: general knowledge and facts and everyday activities. The format of control items matched that of the intervention items (see Figure 3).

### Stage II: Scenario Evaluation

#### Introduction

Before using the items that were created in stage I as training materials for STOP, these items required further validation to ensure their relevance to and suitability for paranoia. Items were rated for paranoia severity and readability, and item length was recorded. We aimed to reduce systematic discrepancies between intervention and control items and between sessions by matching the readability of items and the item length. Matching these aspects across intervention versus control item sets and individual weekly sessions within each set may reduce possible confounding effects. For instance, differences in item comprehension or time spent engaging with each item could inadvertently influence the “dose” of a session. Items were also rated by relevant contributors based on paranoia severity with the aim to distribute intervention such that early training sessions included less severe items, with a graded progression toward more potentially threatening or paranoia severity items in later sessions. On the basis that the training materials could be emotionally triggering for some patients, this graded exposure approach allows patients to progressively work toward more challenging therapeutic content, thereby increasing acceptability and reducing the risk of dropout. Intervention items also consisted of items with higher paranoia severity ratings compared to the control set.

#### Methods

##### Participants

We approached a total of 16 raters; half the raters were a group of living or lived experts independent from those who had created the contents in stage I. Experts were recruited from LEAP and wider networks of The McPin Foundation. The other half of the raters were clinical psychologists recruited from the Psychological Interventions Clinic for outpatients with Psychosis. In total, 15 raters completed all ratings (clinical psychologist: n=7; living experts: n=8), and 1 rater dropped out from being busy after only completing one-third of the ratings. Raters were randomly assigned to rate either intervention (n=8) or control items (n=7). Clinician raters and living or lived experts were reimbursed for their contribution to the study at £50 (US $62.28) and £30 (US $36.67) per hour, respectively. These were the going rates for the relevant experts.

##### Procedures

For the purpose of rating, we included the final word of the passage that completes the text and removed the follow-up yes or no question. For the intervention item, the final word depicted the paranoid interpretation of the ambiguous text. Clinician raters rated the intervention scenarios based on the criteria: paranoia severity and readability. For example, raters were asked to rate the level of paranoia each scenario is likely to evoke (see Multimedia Appendix 1 for additional information on counterbalancing of ratings).

Paranoia severity was rated on a 6-point scale (0=not paranoid; 1=mild paranoia to 5=severe paranoia); readability was rated on a 6-point scale (0=difficult to read; 5=easy to read). A mean rating of ≥1 for the intervention item and ≤1 for the control item was set, a priori, as the acceptable threshold for the severity scale. A mean rating of ≥ 3 was set, a priori, as the acceptable threshold for the readability scale for both experimental conditions. Living or lived experts rated items on the readability criterion only. Paranoia ratings from living experts were not appropriate because to gauge the severity of the potentially paranoid content it was necessary to present items in their negative or paranoid form. This would be a prolonged, unjustifiable, and potentially harmful negative mood induction for these individuals.

Once all data were collected from raters, we conducted an iterative process of reviewing and refining items. First, means were calculated for paranoia severity and readability. Items that fell below the acceptable value were reviewed or replaced (n=43 intervention items did not reach the threshold on the severity scale). These items were discussed among the STOP team, rewritten, and then rerated by the same clinicians (see Multimedia Appendix 1 for interrater reliability data). Finally, three 2-hour Zoom meetings (Zoom Technologies, Inc) were conducted with the members of LEAP (n=4-6) at each meeting to systematically review, item by item, the final intervention and control content. Feedback was recorded, and further minor replacements or revisions were made where essential.

Items were distributed based on paranoia severity, readability, and item length. We evenly distributed intervention items into six, 40-item sessions based on a progression of mean paranoia severity ratings across the 6 sessions (while checking for any discrepancies between readability ratings between intervention and control item sets and between the 6 sessions). Item length—operationally defined by the item’s total character count—was also matched within and between sessions and item sets (see Multimedia Appendix 1 for additional information on cross-referencing of item length).

#### Results

In the first iteration of rating, 24 training items reached acceptable values (paranoia severity: mean 3.48, SD 0.95), all items reached the threshold after rerating (paranoia severity: mean 4.71, SD 0.30). All control items reached the acceptable value for paranoia severity and readability (see Multimedia Appendix 1 for the Analysis Plan).

**Figure 4:**
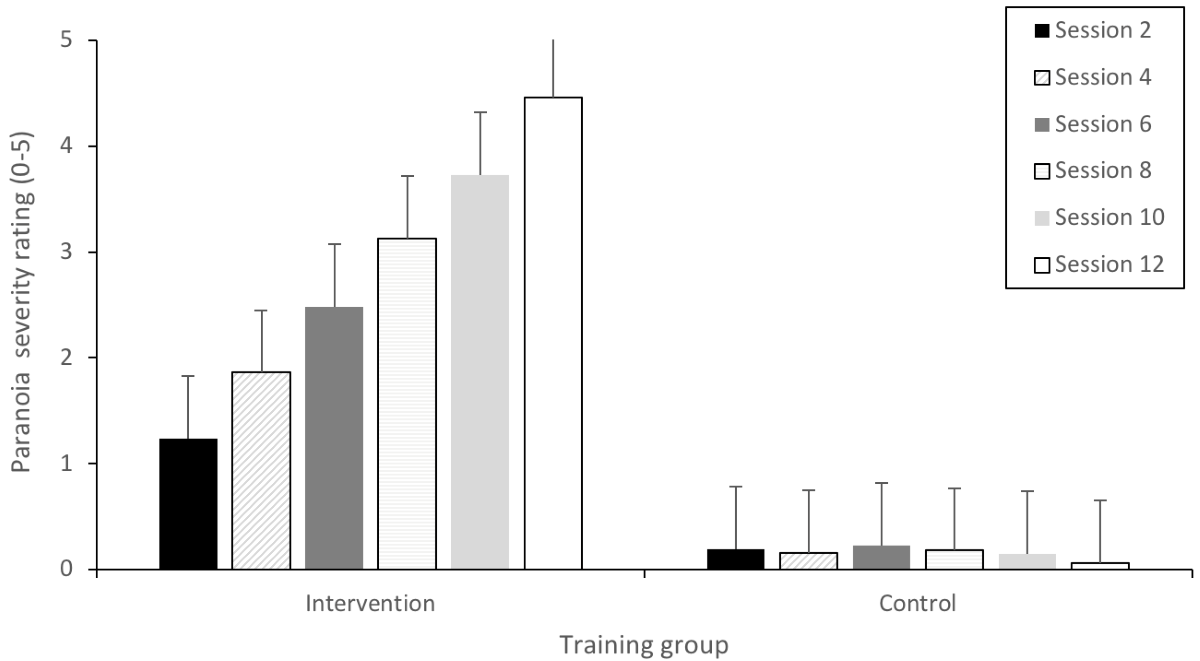
Mean paranoia severity ratings across training groups and sessions.

For item distribution based on paranoia severity, as shown in Table 1, a 2 (intervention and control) × 6 (sessions 7-12) analysis of variance showed a systematic difference in items’ severity between intervention and control item (*F*_1,468_=6201.01; *P*<.001), between sessions (*F*_5,468_=194.76; *P*<.001), and there was an interaction (*F*_5,468_=223.07; *P*<.001). Post hoc examination of the mean severity scores revealed that there was a difference in items’ severity across sessions for the intervention but not the control group (see Figure 4). In STOP, the 6 sessions previously developed as part of the feasibility study [35] were interleaved in addition to the 6 newly created sessions to create 12 sessions based on a progression of mean paranoia severity ratings.

For item length, cross-checking by 3 researchers (MW, TH, and ZYY) showed a high agreement for both intervention (n=223, 93%) and control items (n=227, 94.5%), with (n=480, 100%) agreement between the researchers following resolution. For item distribution based on item length, as shown in Table 1, a 2 (intervention and control) × 6 (sessions 7-12) analysis of variance revealed no systematic differences in the item’s character count between intervention and control items (*F*_1,468_=1.43; *P*=.23), between sessions (*F*_5,468_=0.01; *P*≥.99), and there was no interaction (*F*_5,468_=0.12; *P*=.99).

**Table 1:** Mean (SD) character count and item ratings (intervention and control) of paranoia severity and readability across sessions.

Session	Intervention items, mean (SD)								Control items, mean (SD)					
	Clinician rating (n=8)			User rating (n=8)		Scenario character count		Clinician rating (n=7)				User rating (n=8)		Scenario character count
	Severity	Readability	Readability				Severity			Readability	Readability			
7	1.24 (0.33)	4.17 (0.50)	3.76 (0.60)		153.65 (23.06)		0.19 (0.23)			4.23 (0.51)	3.69 (0.73)		156.62 (41.24)	
8	1.86 (0.47)	4.21 (0.55)	3.73 (0.62)		154.32 (25.18)		0.16 (0.24)			4.21 (0.65)	3.82 (0.63)		155.30 (36.61)	
9	2.48 (0.50)	4.29 (0.48)	3.76 (0.53)		155.07 (30.66)		0.23 (0.28)			3.99 (0.55)	3.50 (0.47)		156.28 (40.69)	
10	3.13 (0.56)	4.46 (0.35)	3.82 (0.57)		151.62 (28.87)		0.18 (0.24)			4.23 (0.52)	3.53 (0.69)		159.28 (34.43)	
11	3.73 (0.57)	4.24 (0.54)	3.76 (0.65)		153.73 (28.13)		0.15 (0.21)			4.13 (0.69)	3.55 (0.75)		158.05 (32.47)	
12	4.46 (0.33)	4.37 (0.38)	4.10 (0.60)		152.95 (29.97)		0.06 (0.15)			4.22 (0.64)	3.56 (0.61)		156.88 (28.84)	
Total	2.82 (1.19)	4.29 (0.48)	3.82 (0.60)		153.56 (27.50)		0.16 (0.23)			4.17 (0.60)	3.61 (0.67)		157.07 (35.63)	

### Stage III: Item Graphics

#### Introduction

In the CBM-pa feasibility trial [35], living or lived experts recommended visually enriching content in addition to text passages to increase the engagement and realism of text scenarios [29]. Indeed, researchers have shown that the effectiveness of CBM clinical interventions is positively correlated with the degree of participants’ active involvement [49]. We, therefore, included graphics to accompany each of the intervention and control items used in STOP.

#### Methods

##### Materials

**Figure 5:**
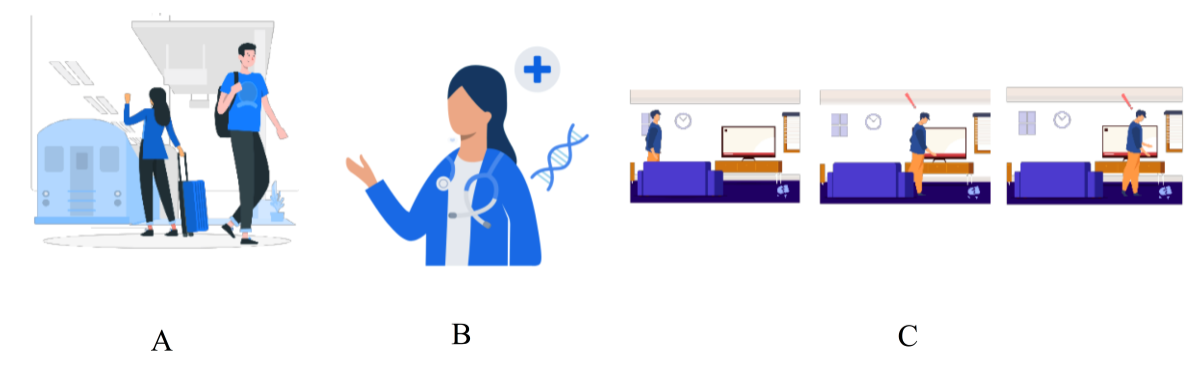
Example of item graphics. (A) Static image, (B) dynamic image, and (C) 3-image scene.

Graphics development was outsourced to an industry partner, Avegen [45]. The STOP research team provided Avegen with text-based scenarios that were developed in the previous stages of this study. Avegen graphics designers created the graphics based on extrapolations of the text-based scenarios. The graphics were chosen to depict the ambiguous scenarios and their nonparanoid interpretation (that runs counter to the paranoid reader’s initial assumption), as well as the neutral control items. Three types of graphics were included (see Figure 5 for an example of each type of graphics) (1) static images (n=576), (2) dynamic images (n=192), and (3) scenes (n=192; each a collection of 3 static images depicting the sequence of events in the unfolding scenario).

##### Participants and Procedure

Once graphics were created, we invited 18 unreimbursed members of the public to rate a random selection (totaling one-quarter of all material) of the graphics used in STOP based on specific attributes of user experience. We randomly selected 25% (n=120) of each type of graphics for the 6 newly created sessions for STOP (total 480 items), and then randomly assigned half of the users (n=9) to rate graphics of intervention items and the other half (n=9) to rate graphics of control items. Participants rated the graphics independently on 3 rating criteria: complexity, attractiveness, and interest, using three 100-point sliding scales (0=the least to 100=the most), 1 for each rating criterion. The 3 rating criteria were selected by 2 researchers (CWH and JY) from 10 scales of the User Experience Questionnaire that described the appearance of interactive products [50]. The 3 rating criteria were selected based on coverage of the scales and their relevance to STOP. At the outset of the graphics rating task, we showed users an example of 2 images on opposite ends of the scales for each rating criterion as anchors. Graphics were presented as a Qualtrics survey with the following instructions:

Welcome to the rating questionnaire. There are 120 items and it should take around 20-30 minutes. Using the sliders, please rate each of the following images against the parameters below.

#### Results

Table 2 shows the ratings on training item graphics as a function of item category (intervention and control). A series of independent samples *t* tests indicated no significant difference between intervention and control graphics across all 3 rating scales (complexity, attractiveness, and interesting).

**Table 2:** User rating on training item graphics (maximum score=100) as a function of item category (intervention and control).

	Intervention items, mean (SD)	Control items, mean (SD)	*t* test (*df*)	*P* value
Complexity	45.64 (22.54)	46.05 (22.80)	0.42 (2158)	.68
Attractiveness	57.72 (19.46)	56.50 (20.26)	1.43 (2158)	.15
Interest	56.90 (20.85)	55.57 (21.19)	1.47 (2158)	.14

### Stage IV: STOP Mobile App Usability Testing

#### Introduction

The STOP app development was outsourced to Avegen [45]. STOP is a mobile app that delivers CBM therapy for paranoia on either Android or iOS platforms. In consultation with the STOP research team, Avegen designed and built the app top-down using the finalized training items developed in the previous stages of this work. STOP provides 1 self-directed weekly therapy session consisting of 40 training items, taking approximately 40 minutes to complete. Users schedule weekly sessions on their STOP phone app, and automatic reminders are sent to users via email before the session. Each item includes user-generated text-based scenarios with accompanying graphics. Session content is interspersed with trivia and badges upon completion of each training session to improve user experience. Living experts are invited to test the STOP phone app and provide feedback during 2 pilot sessions (May and October 2021). Initial aspects of the app design (eg, STOP acronym, logo design, color palette, fonts, layout, storyboard, gamification elements, and instructions for use) were co-designed with the LEAP group (n=4-8) over a period of 6 months through a series of regular group meetings attended by the industry partner and relevant graphic designers. Once the first minimal viable product was achieved, the formal phase of usability testing began.

#### Methods

##### Usability Testing: Participants

A group of living or lived experts (pilot 1: n=5; pilot 2: n=4) separate from those who contributed to the previous stages of this work were recruited by The McPin Foundation as a part of the usability testing for STOP. Again, living experts were reimbursed for their contribution to this study at £30 (US $36.67) per hour.

##### Usability Testing: Procedures

Two piloting sessions of the STOP mobile app were scheduled with living experts to incorporate feedback to refine and improve the product. The first pilot study lasting approximately 45 minutes included a test version of STOP where the content and function of the app were limited, and the second pilot study included the testing of 2 intervention sessions across 2 weeks (from 11 October 2021, to 22 October 2021). In both pilot studies, living experts provided quantitative ratings on the following features of the mobile app: ease of use, user interface, interactive features, design and graphics, security and privacy, errors or bugs, and help provision (see Multimedia Appendix 1 for a description of each feature). These criteria were adapted from the User Experience Questionnaire [50]. Living or lived experts provided a rating of each feature using a 5-point scale (1=inadequate, 2=adequate, 3=good, 4=very good, and 5=excellent). A mean rating of ≥2 was set, a priori, as the acceptable threshold for each scale.

In addition to the ratings described above, in pilot 2, we wanted to understand the kinds of problems or issues users were experiencing and their general experience with the STOP mobile app. As such, we invited users to provide a descriptive account of their experience (eg, “In one or two sentences, describe any problems/issues that you might have encountered when using the App, if any.” “In one or two sentences, describe your overall experience with the App and what you would change, if any”).

#### Results

Table 3 shows the users’ ratings of the STOP mobile app in pilot 1 and pilot 2 (see Multimedia Appendix 1 for users’ descriptive accounts). As shown in Table 3, in both pilots, living or lived experts provided a mean rating above our acceptable threshold for all the evaluated features of the STOP mobile app.

**Table 3:** User ratings of the STOP^a^ mobile phone app (max score=5) from usability testing.

STOP mobile app feature	Pilot 1, mean (SD)	Pilot 2, mean (SD)
Ease of use	4.2 (0.45)	4.25 (0.50)
User interface	4.2 (0.84)	4.5 (0.58)
Interactive features	4.2 (0.84)	4.25 (0.96)
Design and graphics	4.4 (0.55)	5 (0.00)
Help provision	N/A^b^	4 (0.82)
Security and privacy	4.4 (0.55)	5 (0.00)
Errors or bugs	4.4 (0.89)	Descriptive account of any errors or bugs. Any errors identified have been resolved.
Overall experience	4 (0.71)	Descriptive account of overall experience with the app (see Multimedia Appendix 1)

^a^STOP: Successful Treatment of Paranoia.

^b^N/A: not applicable.

### Ethical Considerations

The STOP trial program of work received ethical approval from the London-Stanmore Research Ethics Committee (reference 21/LO/0896), and all those participating in the work described gave consent for publication.

## Discussion

### Principal Findings

This study focused on the development of new material to be used in STOP—a novel mobile phone app designed to reduce the symptoms of paranoia. This self-administered digital therapeutic aims to reduce symptoms by presenting everyday ambiguous situations that can trigger paranoid thoughts and then normalizing users’ interpretations of these situations. However strong the conceptual basis of a new therapeutic, its quality, acceptability, and efficacy will be dependent upon its detailed content, input, and recommendations from various relevant contributors [38,40]. This is especially true for interventions that are based on CBM methods, which rely solely on content for their effect [24,25], and interventions that address psychosis [37,39]. The work presented in this paper represents a 12-month activity with clinicians, living or lived experts, a digital solutions design team, and researchers to develop and evaluate the therapeutic content of the mobile app STOP. Specifically, the co-design approach represents a thorough attempt to achieve our two objectives, which is to (1) take a user-centered approach to create and evaluate paranoia-relevant CBM item content and (2) engage with living or lived experts and the digital solutions design team to create and pilot test the STOP mobile app prototype.

For all training materials, we reached a priori-defined acceptable threshold for all rating criteria: paranoia severity and readability of the scenarios, and there were no systematic differences in item length between intervention and control content nor within the 6 newly created sessions of STOP. These data were used to inform the progression of the therapeutic intervention by arranging session content in order to increase paranoia severity. To reflect clinician-administered cognitive therapies, a “drill-down” approach from surface-level automatic thoughts to more profound core beliefs was adopted across sessions by using selected specific verbs to reflect on each level of thought process. For item graphics, we also found no systematic differences in users’ ratings of complexity, attractiveness, and interest between intervention and control groups. Furthermore, evaluations from 2 pilot tests of STOP with living or lived experts showed that user ratings were above our a priori acceptable thresholds for all evaluated features of the mobile app, suggesting that users found the STOP app easy to use and navigate, suitably interactive, helpful, and secure.

### Comparison With Prior Work

The existing literature demonstrates the importance of co-designing mobile phone apps for mental illnesses with multiple collaborators [37-39]. This work illustrates 1 approach to implementing a detailed user-centered development process that was applied throughout the entire design and development process of a mobile app. This may serve as a useful model for others, as the field of digital mental health continues to grow exponentially. Our co-design is likely to have improved the relevance, authenticity, face validity, and acceptability of both the therapy interface and its content compared to a researcher-led approach, although we cannot provide direct evidence on this. In each phase of STOP’s creation, we involved relevant contributors to provide feedback, open discussion, and formal usability testing of STOP’s content and mobile app. Contrary to STOP’s predecessor CBM-pa [35] where only the researchers designed training materials, in this work, we refined both the therapeutic content (training material) and the mobile app implementation, following contributors’ recommendations. The literature on co-design suggests that the careful and inclusive development process we have followed is likely to enhance user engagement and uptake of STOP [38]. There is also evidence that co-design improves treatment adherence and motivation [51]. Further additional features that we have included, such as graphical enhancement, use of therapeutic content based on actual patient experience, and close attention to the reduction in potential confounding variables (eg, time spent in therapy could inadvertently influence the “dose” of a session), may improve the intervention when tested against a control group in a clinical trial.

### Limitations

There are several improvements that could be made to this study. First, despite basing content development on user-generated examples, the generalization of these examples is limited to the individuals that generated them. Future work should consider ways to tailor content to the individual in real time or prior to the start of therapy. The development of personalized predictive algorithms and agile methods of therapeutic content selection will be one way to do this. Second, it will be important to test the STOP app for acceptability and feasibility of usage in a live clinical service setting, as mentioned by national organizations such as The National Institute for Health and Care Excellence [52]. By the same token, our small sample of raters was recruited from single clinical service units within the United Kingdom, thereby limiting the representativeness of the feedback and ratings received.

Third, there are several limitations relevant specifically to a clinical trial context use of STOP, as opposed to real-world deployment. For example, we only matched items by length between experimental and control groups, as measured by items’ character counts; a more thorough matching process would likely reduce any further confounding effects of the training material. Using single factors such as these to control for arbitrary effects of the intervention is limited, and in the future, other factors could be added to better control for confounds (eg, measuring actual reading speed, user’s comprehension of items, gender-specific content, and intercultural relevance).

A further trial-related limitation is that graphics were rated on only 3 rating criteria pertaining to visual appearance, which were derived from subscales of a standardized instrument. The limited selection of scales was a pragmatic decision, and future work could match graphic content on a wider range of criteria, for example, including aspects of appearance, such as aesthetics, excitement, likeability, and so on, all of which are included in the original instrument that was used to motivate our selection of scales. In addition to graphic enrichments, other elements, including badges, progress trackers, and trivia, are integral to the STOP mobile app and are derived from earlier focus group discussions, but these have not been evaluated. Ideally, all enrichments should be tested systematically to determine their effectiveness in engaging and motivating service users.

Finally, although we rely on feasibility data and previous ratings and feedback [25] to validate the first 6 sessions of STOP, nevertheless, an improvement in future work would be to evaluate all 12 sessions simultaneously on the same metrics.

### Conclusions

In conclusion, CBM-pa is a relatively recent novel psychological intervention that has now been extended into the digital therapeutic called STOP. Material development and app development for any new CBM content should follow an iterative and rigorous process involving multiple contributors that include living or lived experts, researchers, clinicians, and the design team. This user-centered approach to intervention development maximizes the relevance of therapeutic content to the target user group. In so doing, researchers will most likely also optimize user acceptability, effectiveness, and engagement to create the best possible mobile health interventions for people with severe psychiatric disorders.

## Appendix

Multimedia Appendix 1 STOP development.

## Abbreviations

CBM: cognitive bias modification
CBM-pa: Cognitive bias modification for paranoia
CBT: cognitive behavioral therapy
LEAP: Lived Experience Advisory Panel
STOP: Successful Treatment of Paranoia

## References

[1] Freeman D, McManus S, Brugha T, Meltzer H, Jenkins R, Bebbington P (2011). Concomitants of paranoia in the general population. Psychol Med.

[2] Schizophrenia Commission (2012). The abandoned illness: a report from the Schizophrenia Commission. Rethink Mental Illness.

[3] Freeman D, Garety PA, Kuipers E, Fowler D, Bebbington PE (2002). A cognitive model of persecutory delusions. Br J Clin Psychol.

[4] Wessely S, Buchanan A, Reed A, Cutting J, Everitt B, Garety P, Taylor PJ (1993). Acting on delusions. I: prevalence. Br J Psychiatry.

[5] Castle DJ, Phelan M, Wessely S, Murray RM (1994). Which patients with non-affective functional psychosis are not admitted at first psychiatric contact?. Br J Psychiatry.

[6] Johnson J, Horwath E, Weissman MM (1991). The validity of major depression with psychotic features based on a community study. Arch Gen Psychiatry.

[7] Goodwin FK, Jamison KR (2007). Manic-Depressive Illness: Bipolar Disorders and Recurrent Depression.

[8] Hamner MB, Frueh BC, Ulmer HG, Arana GW (1999). Psychotic features and illness severity in combat veterans with chronic posttraumatic stress disorder. Biol Psychiatry.

[9] van Os J, Verdoux H, Maurice-Tison S, Gay B, Liraud F, Salamon R, Bourgeois M (1999). Self-reported psychosis-like symptoms and the continuum of psychosis. Soc Psychiatry Psychiatr Epidemiol.

[10] Appelbaum PS, Robbins PC, Roth LH (1999). Dimensional approach to delusions: comparison across types and diagnoses. Am J Psychiatry.

[11] Mehl S, Werner D, Lincoln TM (2015). Does cognitive behavior therapy for psychosis (CBTp) show a sustainable effect on delusions? A meta-analysis. Front Psychol.

[12] van der Gaag Mark, Valmaggia LR, Smit F (2014). The effects of individually tailored formulation-based cognitive behavioural therapy in auditory hallucinations and delusions: a meta-analysis. Schizophr Res.

[13] Lincoln TM, Peters E (2019). A systematic review and discussion of symptom specific cognitive behavioural approaches to delusions and hallucinations. Schizophr Res.

[14] Freeman D, Emsley R, Diamond R, Collett N, Bold E, Chadwick E, Isham L, Bird JC, Edwards D, Kingdon D, Fitzpatrick R, Kabir T, Waite F (2021). Comparison of a theoretically driven cognitive therapy (the Feeling Safe Programme) with befriending for the treatment of persistent persecutory delusions: a parallel, single-blind, randomised controlled trial. Lancet Psychiatry.

[15] Elkis H (2007). Treatment-resistant schizophrenia. Psychiatr Clin North Am.

[16] Turner DT, van der Gaag M, Karyotaki E, Cuijpers P (2014). Psychological interventions for psychosis: a meta-analysis of comparative outcome studies. Am J Psychiatry.

[17] Moritz S, Woodward TS (2007). Metacognitive training in schizophrenia: from basic research to knowledge translation and intervention. Curr Opin Psychiatry.

[18] Waller H, Freeman D, Jolley S, Dunn G, Garety P (2011). Targeting reasoning biases in delusions: a pilot study of the Maudsley review training programme for individuals with persistent, high conviction delusions. J Behav Ther Exp Psychiatry.

[19] Chen J, Short M, Kemps E (2020). Interpretation bias in social anxiety: a systematic review and meta-analysis. J Affect Disord.

[20] Everaert J, Podina IR, Koster EHW (2017). A comprehensive meta-analysis of interpretation biases in depression. Clin Psychol Rev.

[21] Amin N, Foa EB, Coles ME (1998). Negative interpretation bias in social phobia. Behav Res Ther.

[22] Yiend J, Allen P, Lopez ND, Falkenberg I, Tseng HH, McGuire P (2019). Negative interpretation biases precede the onset of psychosis. Behav Ther.

[23] Trotta A, Kang J, Stahl D, Yiend J (2020). Interpretation bias in paranoia: a systematic review and meta-analysis. Clinical Psychological Science.

[24] Savulich G, Freeman D, Shergill S, Yiend J (2015). Interpretation biases in paranoia. Behav Ther.

[25] Savulich G, Shergill SS, Yiend J (2017). Interpretation biases in clinical paranoia. Clin Psychol Sci.

[26] Cristea IA, Kok RN, Cuijpers P (2015). Efficacy of cognitive bias modification interventions in anxiety and depression: meta-analysis. Br J Psychiatry.

[27] Cristea IA, Kok RN, Cuijpers P (2016). The effectiveness of cognitive bias modification interventions for substance addictions: a meta-analysis. PLoS One.

[28] Jones EB, Sharpe L (2017). Cognitive bias modification: a review of meta-analyses. J Affect Disord.

[29] Leung CJ, Fosuaah A, Frerichs J, Heslin M, Kabir T, Lee TMC, McGuire P, Meek C, Mouchlianitis E, Nath AS, Peters E, Shergill S, Stahl D, Trotta A, Yiend J (2019). A qualitative study of the acceptability of cognitive bias modification for paranoia (CBM-pa) in patients with psychosis. BMC Psychiatry.

[30] Malla AK, Norman RMG, Manchanda R, Townsend L (2002). Symptoms, cognition, treatment adherence and functional outcome in first-episode psychosis. Psychol Med.

[31] Yang R, Cui L, Li F, Xiao J, Zhang Q, Oei TPS (2017). Effects of cognitive bias modification training via smartphones. Front Psychol.

[32] Beard C, Ramadurai R, McHugh RK, Pollak JP, Björgvinsson T (2021). HabitWorks: development of a CBM-I smartphone app to augment and extend acute treatment. Behav Ther.

[33] Steel C, Wykes T, Ruddle A, Smith G, Shah DM, Holmes EA (2010). Can we harness computerised cognitive bias modification to treat anxiety in schizophrenia? A first step highlighting the role of mental imagery. Psychiatry Res.

[34] Turner R, Hoppitt L, Hodgekins J, Wilkinson J, Mackintosh B, Fowler D (2011). Cognitive bias modification in the treatment of social anxiety in early psychosis: a single case series. Behav Cogn Psychother.

[35] Yiend J, Lam CLM, Schmidt N, Crane B, Heslin M, Kabir T, McGuire P, Meek C, Mouchlianitis E, Peters E, Stahl D, Trotta A, Shergill S (2023). Cognitive bias modification for paranoia (CBM-pa): a randomised controlled feasibility study in patients with distressing paranoid beliefs. Psychol Med.

[36] Nilsen W, Kumar S, Shar A, Varoquiers C, Wiley T, Riley WT, Pavel M, Atienza AA (2012). Advancing the science of mHealth. J Health Commun.

[37] Ben-Zeev D, Brenner CJ, Begale M, Duffecy J, Mohr DC, Mueser KT (2014). Feasibility, acceptability, and preliminary efficacy of a smartphone intervention for schizophrenia. Schizophr Bull.

[38] Schouten SE, Kip H, Dekkers T, Deenik J, Beerlage-de Jong N, Ludden GDS, Kelders SM (2022). Best-practices for co-design processes involving people with severe mental illness for eMental health interventions: a qualitative multi-method approach. Des Health.

[39] Kidd SA, Feldcamp L, Adler A, Kaleis L, Wang W, Vichnevetski K, McKenzie K, Voineskos A (2019). Feasibility and outcomes of a multi-function mobile health approach for the schizophrenia spectrum: App4Independence (A4i). PLoS One.

[40] Hsu CW, Akuhata-Huntington Z (2023). Implicit bias training for New Zealand medical students using cognitive bias modification: an outline of material development. New Zealand J Psychol.

[41] Maguire M (2001). Methods to support human-centred design. Int J Hum Comput Stud.

[42] Mathews A, MacLeod C (1994). Cognitive approaches to emotion and emotional disorders. Annu Rev Psychol.

[43] Yiend J, Barnicot K, Williams M, Fox E (2018). The influence of positive and negative affect on emotional attention. J Behav Ther Exp Psychiatry.

[44] Freeman D, Garety PA, Bebbington PE, Smith B, Rollinson R, Fowler D, Kuipers E, Ray K, Dunn G (2005). Psychological investigation of the structure of paranoia in a non-clinical population. Br J Psychiatry.

[45] (2022). Avegen.

[46] Beck AT, Rush AJ, Shaw BF, Emery G (1979). Cognitive Therapy of Depression.

[47] Bobak Carly A, Barr Paul J, O'Malley A James (2018). Estimation of an inter-rater intra-class correlation coefficient that overcomes common assumption violations in the assessment of health measurement scales. BMC Med Res Methodol.

[48] (2018). McPin Foundation.

[49] Hoppitt L, Mathews A, Yiend J, Mackintosh B (2010). Cognitive bias modification: the critical role of active training in modifying emotional responses. Behav Ther.

[50] Laugwitz B, Held T, Schrepp M (2008). Construction and evaluation of a user experience questionnaire.

[51] Killikelly C, He Z, Reeder C, Wykes T (2017). Improving adherence to web-based and mobile technologies for people with psychosis: systematic review of new potential predictors of adherence. JMIR Mhealth Uhealth.

[52] (2019). Evidence standards framework for digital health technologies. National Institute for Health and Care Excellence.

