# Supplementary material for: User-Centered Development of STOP (Successful Treatment for Paranoia): Material Development and Usability Testing for a Digital Therapeutic for Paranoia
Source: JMIR Hum Factors. 2023 Dec 8;10:e45453. doi: 10.2196/45453 (PMC10746980; doi:10.2196/45453)
Supplement: Multimedia Appendix 1 [file humanfactors_v10i1e45453_app1.docx]

**Additional Details of STOP Development**

**STOP Content Design (Test and Images)**

All stages of the development and testing were conducted by the STOP research team and co-led by staff from the McPin Foundation who promote service user input into mental health research. The research team included clinical psychologists, psychiatrists, academics, and psychology master’s and doctorate level students. Inclusion criteria of **living/lived experts** included *1) historical or current first-hand experience of paranoia either personally or through regular contact with another person; 2) age 18+ years; 3) fluency in English.* Inclusion criteria for **clinical psychologists** were *clinicians with a minimum of two years of experience working with patients with paranoia.*

**Stage I: Scenario creation outline**

***Intervention Items.*** Each expert was asked to provide 20 short descriptions of typically encountered everyday ambiguous situations that had the potential to trigger paranoid thoughts and to provide an associated paranoid and non-paranoid interpretation of each.

Scenarios were arranged into six topic areas/categories:

1. Social/Interpersonal threat (e.g., being gossiped about, judged, disliked, dumped).
2. Delusions of reference/magical thinking (e.g., identifying personal messages).
3. Threat of persecution/spying (e.g., being stalked, spied on, followed).
4. General suspiciousness/distrust (e.g., being robbed, plagiarised, irritated by neighbours).
5. Medical/Paramedical/healthcare threat (e.g., being prescribed drugs with adverse

effects, being conspired against by medical staff, having one’s DNA stolen).

6. Physical harm (e.g., being poisoned, injured, threatened by terrorism, killed).

The STOP research team adapted suitable scenarios into intervention items in the format commonly used for CBM training items. Clinician-administered cognitive therapies traditionally employ an increasing 'drill-down' from more surface-level automatic thoughts, into more stable rules and assumptions, and then finally toward deeper core beliefs [1]. To reflect this in our training sessions we selected specific verbs for the final sentence of each passage. For example, in the two early sessions, the following verbs were used to reflect automatic thoughts: ‘think’, ‘imagine’, or ‘sense’ (e.g., you *think* that this means…). For the two mid-sessions, the following verbs were used to capture underlying assumptions: ‘assume’, ‘presume’, ‘suppose’ (e.g., you *assume* that this means …). In the final two sessions, core beliefs were reflected using the verbs ‘believe’, ‘are sure’, ‘know’ (e.g., you *believe* that this means …). Appropriate verbs to use to capture each level were decided by consensus of three members of the research team with experience in paranoia, cognitive therapies, and CBM methods.

**Stage II: Scenario Evaluation**

***Procedures for Counterbalancing Ratings.*** Items were rated in three separate batches of 80 items. For each batch, the presentation order of items (ascending; reversed), and the order in which clinician raters rated each criterion were counterbalanced using a Latin Square design. For example, rater R1 rated the first batch of 80 items in reverse order on readability, then severity, and then rated the second batch of items in ascending order on readability, then severity; rater R2 rated the first batch of 80 items in reverse order on severity, then readability, and then rated the second batch of items in ascending order on severity, then readability.

***Procedures for Cross-referencing Item Length.*** Three researchers from the STOP study calculated and recorded the item length for 160 items (i.e., one-third of the total items) each. One of those researchers cross-checked 30% of items assigned to a different person. Researchers were assigned each cross-check using the Latin Square Design (i.e., R1 cross-checked items for R2; R2 cross-checked items for R3; and R3 cross-checked items for R1). Any discrepancies (i.e., accidentally counting a space) were rechecked and corrected.

***Interrater Reliability Data.*** Reliability between raters on each of the rating criteria for the intervention items were: ICC_severity_ = .84 (clinician raters only), ICC_readability_ = .46, and for the re-rating by the two clinicians (ICC_severity_ = .59). Reliability between raters on each of the rating criteria for the control items showed consistently low ratings for paranoia severity and high ratings for readability, ICC_readability_ = .67 (we do not report an ICC score for ratings on paranoia severity because control items, as might be expected, were at floor on the severity scale, meaning that there was insufficient range in these data to make meaningful interpretations of ICC values [2]).

***Analysis Plan.*** Data were analyzed using a two-way Analysis of Variance (ANOVA) in the IBM Statistical Package of Social Sciences (SPSS) version 29. Prior to analyses, normality was assessed using Shaprio-Wilk test and Levene’s Test was used to assess for homogeneity of variance.

**Stage IV: STOP Mobile App Usability Testing**

***Description of App Features in Usability Testing***

- *Ease of Use* (e.g., How easy was it to learn how to use the App?)
- *User Interface* (e.g., How attractive was the App’s interface?)
- *Interactive Features* (e.g., How interesting were the trivia and badges?)
- *Design and Graphics (e.g.,* How enjoyable did you find the graphics, animation, sound?)
- *Security and Privacy* (e.g., How clear was the privacy instruction?)
- *Errors/ Bugs* (e.g., In one to two sentences, describe any problems/issues that you might have encountered when using the App, if any)
- *‘Help’ provision* (function available only at pilot 2; e.g., How easy was it to locate the help function in the App?).

***Users’ descriptive accounts of the STOP mobile phone app from Pilot 2 usability testing.***

| **STOP Mobile App Features** | **Examples of User Feedback** |
| --- | --- |
| Ease of use | “*The app is very easy to use and it is clear how to navigate around it.”*  “*seamless, no improvements*” |
| User interface | “*The interface made it easy to use the app*.”  “*The app is user friendly with clear navigation*.” |
| Interactive features | “*I think the badges is a great way to view progress within the app*” |
| Design and graphics | “*I really liked the sounds, graphics and animations, I thought they were appropriate and fun.”*  “*I love the design graphics animations and sounds.”* |
| Help provision | “*The help functions seemed good and easy to access.”* |
| Security and privacy | “*The app asked for a pin every time I entered it. It made me feel secure.”* |
| Errors/bugs | “*The biggest issue was not receiving the reminders.”*  “*I have encountered a problem in registering in the app. After entering the alphanumeric code given by the researchers and my mobile number, I should have received a password on my mobile by text. I have never received that text.”* |
| Overall rating | “*I really enjoyed using the app and it did have a game-like feel. It didn't feel like work*”  “*I really like the visual appeal and the scenario content*” |

**Additional References**

[1] Beck, A. T., Rush, A. J., Shaw, B. F., & Emery, G. (1979). *Cognitive Therapy of Depression*. New York: Guildford Press.

[2] Bobak, C, A., Barr, P. J., & O’Malley, A. J. (2018). Estimation of an inter-rater intra-class correlation coefficient that overcomes common assumption violations in the assessment of health measurement scales. *BMC Medical Research Methodology, 18,* Article: 93. PMID: 30208858
